# Intermittent Fasting and the Possible Benefits in Obesity, Diabetes, and Multiple Sclerosis: A Systematic Review of Randomized Clinical Trials

**DOI:** 10.3390/nu13093179

**Published:** 2021-09-13

**Authors:** María Morales-Suarez-Varela, Ester Collado Sánchez, Isabel Peraita-Costa, Agustín Llopis-Morales, José M. Soriano

**Affiliations:** 1 Unit of Preventive Medicine and Public Health, Department of Preventive Medicine and Public Health, Food Sciences, Toxicology and Forensic Medicine, University of Valencia, 46100 Burjassot, Spain; escosan@alumni.uv.es (E.C.S.); ipecos@alumni.uv.es (I.P.-C.); allomo@alumni.uv.es (A.L.-M.); 2 CIBER in Epidemiology and Public Health (CIBERESP), Institute of Health Carlos III, 46980 Madrid, Spain; 3 Food & Health Lab, Institute of Materials Science, University of Valencia, 46980 Paterna, Spain; jose.soriano@uv.es; 4 Joint Research Unit on Endocrinology, Nutrition and Clinical Dietetics, University of Valencia-Health Research Institute La Fe, 46026 Valencia, Spain

**Keywords:** intermittent fasting, exercise, obesity, type 2 diabetes, multiple sclerosis

## Abstract

Intermittent fasting has become popular in recent years and is controversially presented as a possible therapeutic adjunct. A bibliographic review of the literature on intermittent fasting and obesity, diabetes, and multiple sclerosis was carried out. The scientific quality of the methodology and the results obtained were evaluated in pairs. Intermittent fasting has beneficial effects on the lipid profile, and it is associated with weight loss and a modification of the distribution of abdominal fat in people with obesity and type 2 diabetes as well as an improvement in the control of glycemic levels. In patients with multiple sclerosis, the data available are too scarce to draw any firm conclusions, but it does appear that intermittent fasting may be a safe and feasible intervention. However, it is necessary to continue investigating its long-term effects since so far, the studies carried out are small and of short duration.

## 1. Introduction

Intermittent fasting (IF) refers to periods of regular times with a very restricted or no caloric intake, that is, periods of voluntary abstinence from food and liquid intake: methods of energy deprivation [1]. Commonly, it consists of a daily fast of 16 h, a 24-h fast on alternate days, or a 2-day-a-week fast on non-consecutive days. During the fasting period, the consumption of calories frequently varies from 0 to 25% in relation to the regular caloric needs. Consumption on non-fasting days can be ad libitum, limited to a certain composition of the diet, or reserved to achieve a specific caloric intake of up to 125% of regular caloric needs. Intermittent fasting can be done with unrestricted consumption when not fasting or in conjunction with other dietary interventions [2]. Fasting, in general, has been rated since the 1960s as a successful strategy to treat obesity and comorbidities, with additional benefits of fasting other than weight loss having been recently uncovered [3]. IF has gained significant public notoriety in recent years thanks to the media; however, it is important to note that it has been performed for several thousand years for health and religious reasons, such as, for example, during the month of Ramadan [4]. Previous studies on fasting have shown generally positive effects on health; however, the benefits and challenges, mainly acceptance and compliance, of a long-term fasting eating behavior still require further research. IF can well fit in everyday life and may be possibly adopted as a lifelong eating behavior [3].

The leading cause of death in developed countries is obesity, and it will soon surpass tobacco-related deaths. As the WHO states, obesity has tripled worldwide since 1975, affecting 13% of the entire adult population in 2016. A new analysis of the body mass index (BMI) of the United States indicated that by 2030, one in two adults will be obese. At the same time that there is an increase in the prevalence of obesity, there is also a prevalence of other chronic diseases, such as cardiovascular diseases and type 2 diabetes [2,4,5]. Currently, more than 90% of people have type 2 diabetes are overweight or obese. It has been proven that a series of modifiable risk factors favor these pathologies, among which are excessive nutrition and sedentary lifestyle. Physiologically, excessive energy intake increases circulating glucose levels and free fatty acids, thus promoting oxidative stress in skeletal muscle, adipocytes, pancreatic β cells, and hepatocytes, modifying the signal transduction of insulin receptors. It decreases the absorption of glucose in the cells by not being able to take advantage of the glucose; there is an increase in liver glycogenolysis and lipolysis of fat tissue, which counteract the effects of insulin. Adipocytes have a restricted capacity to store excess free fatty acids in plasma, which results in the ectopic deposition of fat in the liver, skeletal muscle, or cardiac muscles, contributing to insulin resistance in these tissues. As diabetes progresses, lipotoxicity and oxidative stress limit the secretory function of β cells and promote their apoptosis, leading to insulin deficiency and consequent hyperglycemia [4]. A key component in reversing these metabolic consequences is weight loss. Established dietary interventions to achieve and maintain a 5% weight loss can improve glycemic control and limit the need for glucose-lowering medications in patients with type 2 diabetes who are overweight or obese [6]. Reducing calories is the key to successful weight loss and improved glycemic control [2], raising discrepancies in the scientific literature on what role intermittent fasting has and whether it can be superior to continuous energy restriction in improving lean body mass retention during weight loss. Today, with the prevalence of type 2 diabetes and obesity increasing worldwide, it is essential that physicians and dietitians know whether IF is a feasible dietary therapy for weight loss in patients with these conditions [4,7,8].

Among the diseases studied in relation to intermittent fasting, we find multiple sclerosis (MS), which is defined as an inflammatory demyelinating disease of the central nervous system characterized by different degrees of damage to axons and neurons, supposedly caused by autoimmune mechanisms. MS affects 2.5 million people worldwide, with a significant personal and socio-economic burden. Clinically, MS can be relapsing-remitting or present a progressive course characterized by accumulation of neurological disability with or without superimposed relapsing activity. The development of the disease is not fully explained by genetic risk factors. Environmental factors, such as some infections, low vitamin D levels, smoking, and obesity, have been linked to an increased risk of MS. Experimental autoimmune encephalomyelitis (EAE) is an animal type of MS that has been essential in the development of various treatments for MS [9]. MS is more common in western countries. Eating habits have been considered as a potential factor that helps the epidemiology of MS. There is an added connection between nutrition and immune-inflammatory responses that is the gut microbiome, where diet plays a critical role. Commensal bacteria found in the gut and their metabolites possess the ability to exert both pro-inflammatory and anti-inflammatory responses by controlling T-cell differentiation and immune responses in the gut. After all, this can have systemic effects and lead to or protect against autoimmune diseases even in the EAE type. The intestinal microbiome in patients with relapsing-remitting MS is modified unlike what happens in healthy controls. At the same time, calorie restriction has powerful anti-inflammatory effects. IF is shown to be a protective factor in the animal model of MS through effects on the intestinal microbiota, with similar changes observed in patients with relapsing MS undergoing short-term IF [9].

Given the scarcity of data regarding the role of IF evaluated globally in the scientific literature, the main objective has been to carry out a bibliographic review to know the role of IF on different diseases, such as obesity, type 2 diabetes, and MS, by identifying the specific benefits that may be derived from such a nutritional intervention that could in turn facilitate the identification of patients that would best benefit from an IF intervention.

## 2. Materials and Methods

**Figure 1:**
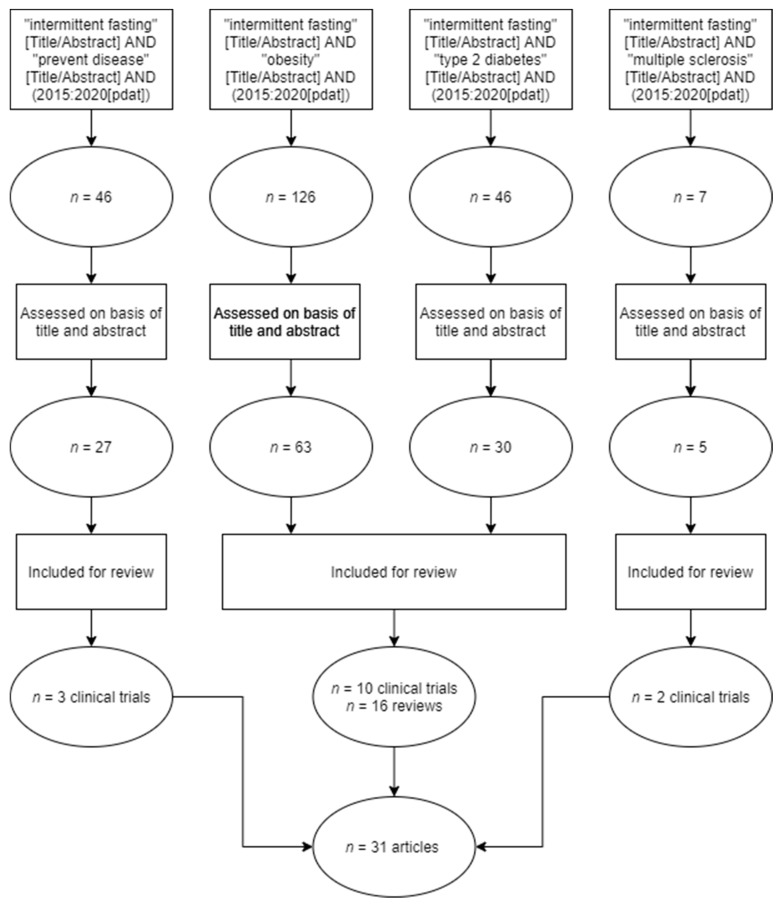
Flow chart of the selection process of articles for the bibliographic review on intermittent fasting and disease prevention.

This systematic review was prepared following the guidelines “Preferred Reporting Items for Systematic Reviews and Meta-Analyzes” (PRISMA) [10,11]; the purpose of these guidelines is to ensure that the articles included in the review are fully reviewed in a clear and comprehensive manner. Transparent, these guidelines use a 27-item checklist detailing the title, abstract, introduction, methods, results, discussion, and funding and also use a four-phase flow chart detailing the inclusion and exclusion of each of the articles presented in Figure 1.

For the present review, searches were carried out in “PubMed” databases of scientific articles. For the correct use of the search terms, the 2015 edition of the descriptors in Health Sciences was consulted on the page http://decs.bvs.br/E/homepagee.htm (accessed on 19 October 2020). An initial search on the effects of IF and the general prevention of disease was carried out. The keywords used to carry out this search were the following: # 1 “intermittent fasting”, # 2 “intermittent fasting” AND “exercise”, # 3 “intermittent fasting” AND “prevent disease”. Similar searches were carried out for # “intermittent fasting”[Title/Abstract] AND “obesity”/”type 2 diabetes”/”multiple sclerosis”[Title/Abstract] limited to articles published between 2015 and 2020. The search was carried out by the authors of the research by reading and synthesizing the information collected and selecting the articles whose content was endowed with greater relevance, specificity, and scientific evidence. The inclusion criteria that have been taken into account are that the articles must have data on the effects of intermittent fasting on obesity, type 2 diabetes, and/or MS and have been randomized controlled trials or reviews published in the last five years (2015–2020).

The selected articles were classified using the scale proposed by the Scottish Intercollegiate Guidelines Network (SIGN) [12] to establish the levels of evidence and the degrees of recommendation of the different studies. The evidence for each work is classified according to its epistemological strength, and only the strongest studies lead to strong recommendations. The scale proposes that the study design and its risk of bias are used to assess the quality of the scientific evidence provided (level of evidence). To assess the study design, numbers (1–4) and signs (+, ++, and -) are used to assess risk of bias. Based on this classification of the evidence in the article, letters (A–D) are used to classify the strength of the recommendations associated with the papers. This scale was used following the principles of evidence-based medicine (EBM), which emphasizes the use of evidence from well-designed studies and well-conducted research. The objective of EBM MBE is that decision-making is based on the most up-to-date, scientifically sound, and reliable evidence for each individualized circumstance that is studied. It should also be clarified that, depending on each specific case, there are certain situations in which it is not possible to obtain the highest levels of evidence due to ethical impediments or other limitations. These restrictions in some cases are insurmountable, so they should not be considered as limitations of the studies but as their own intrinsic characteristics.

## 3. Results

In the initial search on the effects of IF and the general prevention of disease, 46 62 articles were identified in PubMed that met the inclusion criteria. After reviewing these articles, three articles were selected as of interest for this bibliographic review. Of the studies focused on obesity, type 2 diabetes, and MS, 12 (10 randomized clinical trials on the role of intermittent fasting on obesity and diabetes and 2 randomized clinical trials on the role of intermittent fasting on MS) were finally selected alongside the three previously identified randomized clinical trial studies on the effects of intermittent fasting and the general prevention of disease. Furthermore, 16 reviews on the role of intermittent fasting on obesity and diabetes were also identified and included in this review for comparison. The selected studies were analyzed for the following characteristics: year of completion, location, duration of the study, sample size, type of study, subject exposure time, objective of the studies, level of evidence, and the degree of recommendation.

Table 1 shows the characteristics of three articles that identified possible benefits of intermittent fasting for the general prevention of diseases. There were large differences in sample size, but all three were clinical trials, so the level of evidence and the degree of recommendation were high. They all found a positive association between IF and improved health status.

Table 2 shows the ten articles that studied the relationship between IF and obesity and/or diabetes. The sample sizes of these studies ranged from 10 to 250 individuals, and all were randomized clinical trials whose durations ranged from 3 days to 12 months. They all agreed on the benefit of IF in the control of metabolism, specifically in its effects on insulin levels and the cardiometabolic improvement. McAllister et al. [15] evaluated the effects of early feeding with time restriction on insulin sensitivity in the context of type 2 diabetes mellitus. Sutton et al. [16] and Li et al. [17] compared the effect of IF for one week while on a regular diet. Others studied IF in relation to obesity and focused mainly on the effects of IF on weight loss as well as comparing the effect of IF vs. a normal diet on cardiometabolic risk factors and inflammatory markers in adults with obesity [6,17,18,19,20,21,22]. One trial performed a proteomic analysis of human plasma during fasting in people with obesity [23]; one focused on the effects on gene expression, circulating hormones, and diurnal patterns [20]; and another investigated the effects on cognition [24]. The level of evidence and the grade of recommendation were both high for all selected studies.

Table 3 shows the two studies we identified that studied the relationship of IF with MS. Both were carried out in humans, with one of them combining it with an experimental animal study. These trials investigated the feasibility and safety of adopting an IF diet [9] and compared the effects of adopting a ketogenic diet or a fasting diet compared to a conventional diet in patients with MS [25]. Both found a benefit of IF in patients with MS.

Table 4 shows the sixteen literature reviews that previously assessed the role of IF on obesity and diabetes. These reviews focused on the benefits of IF associated with weight loss or muscle gain, the metabolic impact of IF, and the influence of IF on leptin and adiponectin levels.

## 4. Discussion

Both IF and short-term calorie-restricted diets produce similar weight loss in people with obesity and people with type 2 diabetes [17,24,29]. There are few long-term clinical trials, but these have revealed the superiority of IF over caloric restriction in reducing waist circumference and central fat distribution, both of which are beneficial since these parameters are important in reducing cardiovascular risk [13,14,17,19,22,25,26,29]. More long-term studies should be carried out to see how IF affects both obesity and type 2 diabetes [2,13]. Likewise, IF has benefits in slowing the development of atherosclerosis since it inhibits the development of atherosclerotic plaques by reducing the concentrations of inflammatory markers, such as IL-6, homocysteine, and C-reactive protein [28,31]. Some adverse effects of fasting are headaches, which are helped by water intake, bad mood, slight dizziness, and fatigue [22,28].

IF has an important role in the regulation of the levels of different proteins, such as apolipoprotein A4 (APOA4). High levels of APOA4 have been associated with many beneficial phenotypes, such as the promotion of reverse cholesterol transport, increased satiety, and decreased oxidation of LDL-cholesterol particles. IF also has a combined regulatory effect that increases triglyceride lipolysis in chylomicrons and increases the production of mature spherical HDL-cholesterol particles [21,23], thus producing a significant decrease in total triglyceride levels in plasma after a period of IF. However, no significant differences were observed in HDL-cholesterol levels after IF, suggesting more trials with more participants are needed to validate these findings [13,20,32]. In another trial of early time-restricted feeding (eTRF), both LDL-cholesterol and HDL-cholesterol increased in the morning, which can be attributed to the period of prolonged fasting. The metabolism of these people had a greater dependence on fat oxidation [21,33].

Time-restricted feeding (TRF), which is a new form of IF that improves cardiometabolic health, might slow tumor progression, delay ageing, and increase life expectancy in rodents. Pilot studies in humans similarly suggest that TRF improves clinical outcomes, such as body weight, blood pressure, and insulin sensitivity [8,13,14,15,16,17,23,27,28,30], at least when food intake is limited to the beginning or to the middle of the day [21,23]. These effects related to the time of day can be explained by the circadian system, since eating in accordance with the circadian rhythms of our metabolism can have benefits on cardiometabolic health [16,21,30,31]. One clinical trial found that eTRF decreased fasting glucose and insulin levels in the morning, increased fasting insulin at night, and decreased 24-h blood glucose peaks. The decrease in the 24-h glucose peak was unexpected since the maximum level of postprandial glucose was expected to be higher in the eTRF group; the meals had been consumed in a short time. A possible explanation for this decrease was found, especially at lunchtime: it was possible that circulating insulin was still elevated because breakfast had been eaten relatively recently and was still being digested. For this reason, it was not necessary for pancreatic β cells to be activated again to secrete insulin, which resulted in a smaller plasma glucose peak. This relationship of condensing meals to within a shorter stretch of each day is beneficial to the control of 24-h blood glucose levels regardless of the circadian rhythm. This is evidence that should be further investigated in future clinical trials since it could be very effective at controlling 24-h blood glucose levels [16,17,21]. In contrast, IF in this situation would be less effective for the control of 24-h blood glucose since the periods of time between meals are longer. However, IF is not the same as a lower frequency of meals; the relationship between both variables should be studied in greater depth in the future [21].

It has also been observed that eTRF increased β-hydroxybutyrate in the morning in relation to the control diet, which demonstrated that even in short periods of fasting, circulating ketones can be moderately increased [8,14,21]. High levels of ketones reduce oxidative stress [8,15,21], preserve lean mass [27,28], and have other metabolic effects, such as decreased hunger. It has been suggested in previous studies that eTRF may increase amplitude of the cortisol rhythm, providing a mechanism through which meal timing may impact the circadian system [21]. Hormones and genes related to longevity and autophagy, such as brain-derived neurotrophic factor (BDNF), sirtuin 1 (SIRT1), and LC3A, have also been shown to be favorably affected during a period of eTRF [21]. These important findings suggested that eTRF could perhaps positively influence cardiometabolic health, may impact the central circadian rhythms, and may have antiaging effects [21,34].

In rodents, IF has a positive impact on adult hippocampal neurogenesis (AHN). IF cannot only improve AHN in healthy mice but also protect against neuronal loss caused by excitotoxic damage in the hippocampus, with a greater survival of neurons than observed in mice fed ad libitum. IF attenuates the decrease in locomotion related to age and disease and the decrease in exploratory behavior and memory retention in mouse models of Alzheimer’s disease, which also demonstrates neuroprotective benefits [23]. Energy restriction can affect the expression of genes involved in neurogenesis and increase the proliferation of neural cells. Low-energy diets during adolescence increase cell proliferation and the number of neurons in the hippocampus and can result in better cognition in adulthood. The restriction of energy intake and not the composition of the diet is important for preventing learning and memory deficits. More human trials should be performed to clarify these results.

IF has neuroprotective and anti-inflammatory effects, which were demonstrated in animal models of stroke and systemic infection as well as in humans with systemic inflammatory conditions. However, few trials have confirmed the effects of IF on immune function or autoimmune diseases such as multiple sclerosis. In mice, IF reduces inflammation, demyelination, and axonal damage in EAE. The immune cells of the peripheral lymph nodes reduce the antigen-specific immune responses and the production of proinflammatory cytokines [9]. Microbiota transplantation from mice that underwent IF to immunized recipient mice produced a reduction in the proliferation of myelin antigen-specific lymphocytes and in the severity of EAE in recipient mice fed a standard diet. These findings lead us to believe that the modifications produced by IF have a beneficial effect on the intestinal microbiome of mice, since they can intercede in the systemic immunomodulatory response to myelin antigens in vivo [9]. In another study on mice, IF delayed the onset of disease, led to a lower severity of the disease, and promoted a decrease in the important markers of inflammation and metabolic hormones, such as leptin [25].

Given this, it could be speculated that since IF may produce an increase in bacterial richness and is also associated with a decrease leptin, bacterial richness and circulating levels of leptin could be negatively correlated. These data confirm what has already been observed in other studies: lower richness of the intestinal microbiota is associated with higher serum levels of leptin and lower levels of adiponectin, in accordance with a systemic proinflammatory phenotype. IF has a surprising effect on the composition of the intestinal microbiota, with the enrichment of the Bacteroidaceae, Lactobacillaceae, and Prevotellaceae families. In EAE, changes in the intestinal microbiota or its metabolites can modulate inflammation and demyelination. Of special importance was the abundance of lactobacilli caused by intermittent fasting, which are commonly used as probiotics given their positive effects, including a decrease in inflammatory immune responses [9]. Together, the above findings demonstrate beneficial changes in the metabolism and in the intestinal microbiome of mice and humans with multiple sclerosis who intermittently fast [9]. The human studies have had limitations, such as the sample size or the short experimental period. For this reason, IF should continue to be studied in more patients over longer periods to corroborate what has been observed thus far [9].

Trials in healthy men and patients with multiple sclerosis have shown that IF can significantly improve depression scores, suggesting an “antidepressant-like” effect, potentially through upregulation of AHN, which has an important role in mood modulation [23,25].

We believe that our review presents a summary of the specific benefits that may be derived from an IF nutritional intervention in patients with chronic diseases, such as obesity, type 2 diabetes, and MS, that may be useful when considering possible treatments in clinical practice as long as it is from a perspective of adequate and personalized control for each patient.

## 5. Conclusions

A consensus was identified in the reviewed literature on the role of intermittent fasting, with a superior effect to diets with caloric restriction with respect to waist circumference and central fat distribution, and beneficial data in reducing cardiovascular risk in people with obesity or diabetes mellitus. IF is identified as having an important role in regulating the levels of different proteins of lipid metabolism. Likewise, it increases triglyceride lipolysis in chylomicrons and increases the production of HDL-cholesterol particles, and this causes the levels of total triglycerides in plasma to decrease significantly. eTRF is beneficial for the 24-h control of glycemic levels of people with diabetes. It also increases β-hydroxybutyrate, which causes ketone levels to rise, and oxidative stress is reduced, lean mass is preserved, and the feeling of hunger decreases. Additionally, IF modifies the composition of the intestinal microbiota, enriching the Bacteroidaceae, Lactobacillaceae, and Prevotellaceae families. The abundance of lactobacilli brought about by IF has positive effects, such as lowering inflammatory immune responses, helping to treat people with MS.

## Figures and Tables

**Table 1:** Summary of identified randomized clinical trials on intermittent fasting (IF).

Authors, Year and Country	Study Duration	Sample Size	Type of Study	Exhibition Time	The Purpose of the Study	Main Effects Observed	LE	GR
Washburn et al., 2019 [13] Utah, USA	-	*n* = 30	Randomized clinical trial	2 days	To elucidate some of the mechanisms for the different benefits attributed to IF in relation to disease, through secondary analyzes of fasting and longevity gene expression during food abstinence trial	A decrease in mean trimethylamine N-oxide was observed, although levels returned to baseline on refeeding. Acute alterations in levels of proline, tyrosine, galactitol, and urea plasma levels were observed along with changes in 24 other metabolites during the fasting period.	1++	A
Stekovic et al., 2019 [14] Graz, Austria	2015–2017	*n* = 60	Randomized clinical trial	4 weeks	To clarify to what extent IF influences human physiology in healthy individuals both in the short and long term	Improved cardiovascular markers, reduced fat mass, and increased β-hydroxybutyrate. On fasting days, the pro-aging amino-acid methionine, among others, was periodically depleted, while polyunsaturated fatty acids were elevated. Reduced levels of age-associated inflammatory marker, low-density lipoprotein, and triiodothyronine.	1++	A
McAllister et al., 2019 [15] Texas, USA	-	*n* = 23 22 patients finished the trial	Randomized clinical trial	28 days	To examine the impact of time-restricted eating on markers of cardio-metabolic health and antioxidant status and to determine whether adaptations of this diet would differ ad libitum compared to isocaloric conditions.	IF was associated with significant reductions in body fat, blood pressure, and significant increases in adiponectin and HDL-cholesterol. No changes in caloric intake were detected.	1++	A

Articles were classified using the scale proposed by the Scottish Intercollegiate Guidelines Network. LE: Level of evidence, GR: Grade of recommendations.

**Table 2:** Studies identified on the role of intermittent fasting (IF) on obesity and diabetes.

Authors, Year and Country	Study Duration	Sample Size	Type of Study	Exhibition Time	The Purpose of the Study	Main Effects Observed	LE	GR
Sutton et al., 2018 [16] Los Angeles, USA	2013–2016	*n* =12 8 patients finished the trial	Randomized clinical trial	5 weeks	To find out the effects of early time-restricted feeding on insulin sensitivity, blood pressure, and oxidative stress in men with prediabetes.	Improved insulin sensitivity, β-cell responsiveness, blood pressure, oxidative stress, and appetite.	1++	A
Li et al., 2017 [17] Berlin, Germany	2015	*n* = 46 36 patients finished the trial	Randomized clinical trial	7 days	To investigate the effects of a one-week fasting period compared to usual care in type 2 diabetes mellitus using a pilot trial.	Decreased mean weight, reduction of abdominal circumference, decrease of systolic/diastolic blood pressure, and increased quality of life. No improvement in HbA1c, insulin, and Homeostatic Model Assessment for Insulin Resistance index.	1++	A
Antoni et al., 2016 [18] Guilford, UK	-	*n* = 14 10 patients finished the trial	Randomized clinical trial	3 days	To investigate the early metabolic response to various degrees of energy restriction, which occurs acutely and before weight loss.	Increased postprandial glucose responses, reductions in postprandial triacylglycerol responses, and 3-day energy intake deficits.	1++	A
Sundfør et al., 2018 [19] Oslo, Norway	2015–2017	*n* = 112	Randomized clinical trial	12 months	To compare the effects of intermittent versus continuous energy restriction in relation to weight loss, maintenance, and cardiometabolic risk factors in adults with abdominal obesity and ≥1 additional component of metabolic syndrome.	Improvement in weight loss, maintenance, and cardiovascular risk factors (waist circumference, blood pressure, triglycerides, and HDL-cholesterol) after one year but with no differences between intermittent and continuous energy restriction.	1++	A
Harney et al., 2019 [23] Adelaida, Australia	2013–2015	*n* = 88 85 patients finished the trial	Randomized clinical trial	10 weeks (2 weeks with normal diet + 8 weeks with intermittent fasting)	To perform a proteomic analysis of human plasma during IF in sedentary people.	Increased apolipoprotein A4 and clusterin and decreased apolipoprotein C2, apolipoprotein A2, C3, and plasma triglycerides.	1++	A
Jamshed et al., 2019 [20] Birmingham, USA	-	*n* = 11	Randomized clinical trial	4 days	To determine how time-restricted feeding affects gene expression, circulating hormones, and diurnal patterns in cardiometabolic risk factors in humans.	Decreased mean 24-h glucose and glycemic excursions, altered lipid metabolism, and circadian clock gene expression.	1++	A
Anton et al., 2019 [21] Florida USA	-	*n* = 10	Randomized clinical trial	4 weeks	To assess the safety and feasibility of time-restricted feeding in an overweight sedentary older adult population.	Decreased body weight, no significant changes in other outcome (waist circumference, cognitive and physical function, health-related quality of life, and adverse events) except for clinically meaningful changes in walking speed and improvements in quality of life, with few reported adverse events.	1++	A
Liu et al., 2019 [6] Adelaida, Australia	-	*n* = 76	Randomized clinical trial	8 weeks	To compare the effects of daily caloric restriction vs. IF on markers of inflammation and extracellular matrix deposition in adipose tissue and skeletal muscle in a controlled feeding trial in overweight or obese women.	Markers of inflammation in serum, subcutaneous adipose tissue, and skeletal muscle were unchanged after fed days. After fasting, non-esterified fatty acids (NEFA), M1-macrophages in adipose tissue and M2-macrophages in muscle were increased, and the changes in NEFA and mRNA of pan-macrophage marker CD68 in adipose tissue were positively correlated.	1++	A
Kim et al., 2020 [24] London, UK	2016	*n* = 43	Randomized clinical trial	4 weeks	To investigate the effects of intermittent and continuous energy restriction on cognition related to the neurogenesis of the human hippocampus.	Significantly improved pattern separation and significant deterioration in recognition memory.	1++	A
Jospe et al., 2020 [22] Dunedin, New Zealand	2014–2015	*n* = 250	Randomized clinical trial	12 weeks	To investigate the implication of dietary intake, weight loss, and metabolic outcomes in overweight adults who could choose to follow Mediterranean diets, intermittent fasting, and standard exercise or high-intensity interval training (HIIT) programs.	Weight loss and reduced systolic blood pressure.	1++	A

Articles were classified using the scale proposed by the Scottish Intercollegiate Guidelines Network. LE: Level of evidence, GR: Grade of recommendations.

**Table 3:** Studies identified on intermittent fasting (IF) and effects on multiple sclerosis (MS).

Authors, Year and Country	Study Duration	Sample Size	Type of Study	Exhibition Time	The Purpose of the Study	Main Effects Observed	LE	GR
Cignarella et al., 2018 [9] Washington, USA	2014–2016	Animal study: 10 mice. Study with patients: 17 subjects, and 1 did not complete it	Randomized clinical trial	In mice (4 weeks) In patients (2 weeks)	To assess the safety, feasibility, and compliance of IF in patients with MS.	Altered blood adipokines and the gut flora resembling protective changes observed in mice.	1++	A
Fitzgerald et al., 2018 [25] Baltimore, USA	2015–2016	*n* = 36 31 patients finished the trial	Randomized clinical trial	8 weeks	To assess the safety and feasibility of different types of diets related to IF in patients with MS.	Weight loss and significant improvements in emotional well-being/depression scores relative to control.	1++	A

Articles were classified using the scale proposed by the Scottish Intercollegiate Guidelines Network. LE: Level of evidence, GR: Grade of recommendations.

**Table 4:** Reviews identified to assess the role of intermittent fasting (IF) on obesity and diabetes.

Authors, Year and Country	Type of Study	Analyzed Study	The Purpose of the Study	Main Effects Observed	LE	GR
Golbidi et al., 2017 [7] Vancouver, Canada	Review	Non-analytical studies, such as a case report or case series	To review different mechanisms related to fasting, aimed at controlling metabolic diseases, particularly diabetes.	IF is associated increased transcription of stress-induced proteins, cellular autophagy, reduced advance glycation end-products, increased adiponectin levels, reduced adipocyte size, lower body weight, and better diabetes control.	3	D
Harris et al., 2018 [26] Glasgow, UK	Systematic review with meta-analysis	Randomized clinical trials or randomized clinical trials with high risk of bias	To examine the effectiveness of IF in treating overweight and obesity in adults compared to usual care treatment or no treatment.	Intermittent energy restriction was more effective than no treatment for weight loss although no significant difference in weight loss was observed in comparison to continuous energy restriction.	1−	A
Zubrzycki et al., 2018 [27] Gdansk, Poland	Systematic review	Non-analytical studies, such as a case report or case series	To find out the role of low-calorie diets and IF in the treatment of obesity and type 2 diabetes.	Low-calorie diets and IF in patients with obesity (including those with coexisting type 2 diabetes) can lead to a reduction in body fat mass and metabolic parameter improvements.	3	D
Malinowski et al., 2019 [28] Bidgostia, Poland	Review	Non-analytical studies, such as a case report or case series	To study the effects of IF on the cardiovascular system, including the progression of atherosclerosis, the benefits for type 2 diabetes mellitus, a decrease in blood pressure, and the exploration of other cardiovascular risk factors (such as lipid profile and inflammation).	IF inhibits the development of atherosclerotic plaque. The IF diet causes an increase of brain-derived neurotrophic factor (BDNF), which results in lowering the systolic and diastolic blood pressure by activating the parasympathetic system. The reduced amount of food consumed when using the IF diet results in a decrease in body weight. It also improves glucose metabolism and increases the sensitivity of tissues to insulin by increasing the β cells of the pancreatic islets. The IF diet also limits cardiac hypertrophy.	3	D
Welton et al., 2020 [2] Canada	Systematic review	Randomized clinical trials or randomized clinical trials with low risk of bias	To examine the evidence for intermittent fasting, an alternative to calorie restricted diets, in the treatment of obesity.	Weight loss of 0.8% to 13.0% of baseline weight with no serious adverse events and improved glycemic control.	1+	A
Correia et al., 2020 [1] Lisboa, Portugal	Systematic review with meta-analysis	Randomized clinical trials or randomized clinical trials with low risk of bias	To summarize the current evidence on the interaction between IF during Ramadan vs time-restricted eating and specific physical performance parameters, such as VO_2max_, vertical jump height, distance in 30 min of running, power output. Wingate maximum and mean.	Maximum oxygen uptake is significantly enhanced with time-restricted eating but reduced with Ramadan intermittent fasting. Additional effects may be observed in body mass and fat mass. Non-significant effects were observed for muscle strength and anaerobic capacity. While Ramadan may lead to impairments in aerobic capacity, time-restricted eating may be effective for improving it.	1+	A
Vitale et al., 2020 [4] Michigan, USA	Systematic review	Randomized clinical trials or randomized clinical trials with low risk of bias	To assess the effects of IF on glycemic control and body composition in adults with obesity and type 2 diabetes.	Strong evidence to support IF as a feasible diet to improve glycemia and body composition measures within 12–24 weeks. Follow-up 12–18 months after IF did not show promising results for continued weight loss and improved glycemic control.	1+	A
Park et al., 2020 [29] Anyang, South Korea	Systematic review with meta-analysis	Randomized clinical trials or randomized clinical trials with low risk of bias	To assess the effects of IF on obesity-related factors and cardiometabolic risk factors in adults.	Significant improvements in BMI, body weight, body fat mass, and total cholesterol.	1+	A
Waldman et al., 2020 [30] Alabama, USA	Review	Non-analytical studies, such as a case report or case series	To research the benefits of time-restricted eating in relation to the prevention of cardiometabolic diseases in high-stress occupations, such as police, firefighters, and the military.	The timing of the feeding-fasting window, with feeding taking place in the waking hours and fasting in the evening hours, might offer the greatest benefit for improving cardiometabolic markers.	3	D
Wang et al., 2020 [31] Zhejiang, China	Systematic review with meta-analysis	Randomized clinical trials or randomized clinical trials with high risk of bias	To assess the effects of IF or diets with dietary restriction on plasma concentrations of inflammatory biomarkers.	Significantly reduced C-reactive protein concentrations but did not significantly reduce tumor necrosis factor-α and interleukin-6 concentrations.	1−	A
Meng et al., 2020 [32] Shandog, China	Systematic review with meta-analysis	Randomized clinical trials or randomized clinical trials with high risk of bias	To summarize the effects of controlled clinical trials examining the influence of IF and dietary restriction diets on lipid profiles.	Significant changes in total cholesterol, low-density lipoprotein cholesterol, and triacylglycerols concentrations and no change in high-density lipoprotein cholesterol.	1−	A
Pureza et al., 2020 [33] São Paulo, Brasil	Systematic review with meta-analysis	Randomized clinical trials or randomized clinical trials with high risk of bias	To assess the effect of early time-restricted feeding (eTRF) on the metabolic profile of overweight adults.	Significant effects on the fasting blood glucose and Homeostatic Model Assessment for Insulin Resistance index.	1−	A
Hawley et al., 2020 [5] Melbourne, Australia	Review	Non-analytical studies, such as a case report or case series	To find out the effects of chrononutrition for the prevention and treatment of obesity and type 2 diabetes in mice and humans.	While data from studies of time-restricted eating in animals and a small number of clinical populations are encouraging, there is a need for multicenter, randomized, clinical trials of comparisons between different eating patterns across a range of human cohorts to determine the most efficacious intervention.	3	D
Borgundvaag et al., 2020 [8] Toronto, Canada	Systematic review with meta-analysis	Randomized clinical trials or randomized clinical trials with high risk of bias	To assess the metabolic impact of IF compared to a standard diet in patients with type 2 diabetes mellitus.	IF induced a greater decrease in body weight especially in heavier patients and in studies of shorter duration but was not associated with reduction in HbA1c.	1−	A
Asif et al., 2020 [34] Ottawa, Canada	Review	Non-analytical studies, such as a case report or case series	To review the epigenetic changes implicated in both metabolic diseases and dietary interventions in primary metabolic tissues (i.e., adipose tissue, liver, and pancreas) with the hope of pinpointing potential biomarkers and therapeutic targets for prevention and treatment of disease.	The global metabolic changes seen in several tissues upon disease and dietary intervention make it difficult to not only characterize the adaptive or pathological role of these epigenetic events but to also pinpoint the primary insult that triggers secondary, systemic aspects of these responses.	3	D
Adafer et al., 2020 [35] Lille, France	Systematic review	Randomized clinical trials or randomized clinical trials with high risk of bias	To review how time-restricted eating affects human health.	Decreased body weight and fat mass with beneficial metabolic effects independent of weight loss.	1−	A

Articles were classified using the scale proposed by the Scottish Intercollegiate Guidelines Network. LE: Level of evidence, GR: Grade of recommendations.

## Data Availability

Not applicable.

## References

[1] Correia J.M., Santos I., Pezarat-Correia P., Minderico C., Mendonca G.V. (2020). Effects of intermittent fasting on specific exercise performance outcomes: A systematic review including meta-analysis. Nutrients.

[2] Welton S., Minty R., O’Driscoll T., Willms H., Poirier D., Madden S., Kelly L. (2020). Intermittent fasting and weight loss: Systematic review. Can. Fam. Physician.

[3] Wilhelmi de Toledo F., Grundler F., Sirtori C.R., Ruscica M. (2020). Unravelling the health effects of fasting: A long road from obesity treatment to healthy life span increase and improved cognition. Ann. Med..

[4] Vitale R., Kim Y. (2020). The effects of intermittent fasting on glycemic control and body composition in adults with obesity and type 2 diabetes: A systematic review. Metab. Syndr. Relat. Disord.

[5] Hawley J.A., Sassone-Corsi P., Zierath J.R. (2020). Chrono-nutrition for the prevention and treatment of obesity and type 2 diabetes: From mice to men. Diabetologia.

[6] Liu B., Hutchison A.T., Thompson C.H., Lange K., Heilbronn L.K. (2019). Markers of adipose tissue inflammation are transiently elevated during intermittent fasting in women who are overweight or obese. Obes. Res. Clin. Pract..

[7] Golbidi S., Daiber A., Korac B., Li H., Essop M.F., Laher I. (2017). Health benefits of fasting and caloric restriction. Curr. Diab. Rep..

[8] Borgundvaag E., Mak J., Kramer C.K. (2021). Metabolic impact of intermittent fasting in patients with type 2 diabetes mellitus: A systematic review and meta-analysis of interventional studies. J. Clin. Endocrinol. Metab..

[9] Cignarella F., Cantoni C., Ghezzi L., Salter A., Dorsett Y., Chen L., Phillips D., Weinstock G.M., Fontana L., Cross A.H. (2018). Intermittent fasting confers protection in CNS autoimmunity by altering the gut microbiota. Cell Metab..

[10] Moher D., Liberati A., Tetzlaff J., Altman D.G. (2009). Preferred reporting items for systematic reviews and meta-analyses: The PRISMA statement. Ann. Intern. Med..

[11] Moher D., Shamseer L., Clarke M., Ghersi D., Liberati A., Petticrew M., Shekelle P., Stewart L.A., PRISMA-P Group (2015). Preferred reporting items for systematic review and meta-analysis protocols (PRISMA-P) 2015 statement. Syst. Rev..

[12] Scottish Intercollegiate Guidelines Network (SIGN) (2015). SIGN 50: A Guideline Developer’s Handbook.

[13] Washburn R.L., Cox J.E., Muhlestein J.B., May H.T., Carlquist J.F., Le V.T., Anderson J.L., Horne B.D. (2019). Pilot study of novel intermittent fasting effects on metabolomic and trimethylamine N-oxide changes during 24-hour water-only fasting in the FEELGOOD Trial. Nutrients.

[14] Stekovic S., Hofer S.J., Tripolt N., Aon M.A., Royer P., Pein L., Madeo F. (2019). Alternate day fasting improves physiological and molecular markers of aging in healthy, non-obese humans. Cell Metab..

[15] McAllister M.J., Pigg B.L., Renteria L.I., Waldman H.S. (2020). Time-restricted feeding improves markers of cardiometabolic health in physically active college-age men: A 4-week randomized pre-post pilot study. Nutr. Res..

[16] Sutton E.F., Beyl R., Early K.S., Cefalu W.T., Ravussin E., Peterson C.M. (2018). Early time-restricted feeding improves insulin sensitivity, blood pressure, and oxidative stress even without weight loss in men with prediabetes. Cell Metab..

[17] Li C., Sadraie B., Steckhan N., Kessler C., Stange R., Jeitler M., Michalsen A. (2017). Effects of a one-week fasting therapy in patients with type-2 diabetes mellitus and metabolic syndrome–A randomized controlled explorative study. Exp. Clin. Endocrinol. Diabetes.

[18] Antoni R., Johnston K.L., Collins A.L., Robertson M.D. (2016). Investigation into the acute effects of total and partial energy restriction on postprandial metabolism among overweight/obese participants. Br. J. Nutr..

[19] Sundfør T., Svendsen M., Tonstad S. (2018). Effect of intermittent versus continuous energy restriction on weight loss, maintenance and cardiometabolic risk: A randomized 1-year trial. Nutr. Metab. Cardiovasc. Dis..

[20] Jamshed H., Beyl R.A., Della Manna D.L., Yang E.S., Ravussin E., Peterson C.M. (2019). Early time-restricted feeding improves 24-hour glucose levels and affects markers of the circadian clock, aging, and autophagy in humans. Nutrients.

[21] Anton S.D., Lee S.A., Donahoo W.T., McLaren C., Manini T., Leeuwenburgh C., Pahor M. (2019). The Effects of Time Restricted Feeding on Overweight, Older Adults: A Pilot Study. Nutrients.

[22] Jospe M.R., Roy M., Brown R.C., Haszard J.J., Meredith-Jones K., Fangupo L.J., Osborne H., Fleming E.A., Taylor R.W. (2020). Intermittent fasting, paleolithic, or mediterranean diets in the real world: Exploratory secondary analyses of a weight-loss trial that included choice of diet and exercise. Am. J. Clin. Nutr..

[23] Harney D.J., Hutchison A.T., Hatchwell L., Humphrey S.J., James D.E., Hocking S., Heilbronn L.K., Larance M. (2019). Proteomic analysis of human plasma during intermittent fasting. J. Proteome Res..

[24] Kim C., Pinto A.M., Bordoli C., Buckner L.P., Kaplan P.C., del Arenal I.M., Jeffcock E.J., Hall W.L., Thuret S. (2020). Energy Restriction Enhances Adult Hippocampal Neurogenesis-Associated Memory after Four Weeks in an Adult Human Population with Central Obesity; a Randomized Controlled Trial. Nutrients.

[25] Fitzgerald K.C., Vizthum D., Henry-Barron B., Vizthum D., Henry-Barron B., Schweitzer A., Cassard S.D., Kossoff E., Hartman A.L., Kapogiannis D. (2018). Effect of intermittent vs. daily calorie restriction on changes in weight and patient-reported outcomes in people with multiple sclerosis. Mult. Scler. Relat. Dis..

[26] Harris L., Hamilton S., Azevedo L.B., Olajide J., De Brún C., Waller G., Whittaker V., Sharp T., Lean M., Hankey C. (2018). Intermittent fasting interventions for treatment of overweight and obesity in adults: A systematic review and meta-analysis. JBI Database Syst. Rev. Implement. Rep..

[27] Zubrzycki A., Cierpka-Kmiec K., Kmiec Z., Wronska A. (2018). The role of low-calorie diets and intermittent fasting in the treatment of obesity and type-2 diabetes. J. Physiol. Pharmacol..

[28] Malinowski B., Zalewska K., Węsierska A., Sokołowska M.M., Socha M., Liczner G., Pawlak-Osińska K., Wiciński M. (2019). Intermittent fasting in cardiovascular disorders—An overview. Nutrients.

[29] Park J., Seo Y.G., Paek Y.J., Song H.J., Park K.H., Noh H.M. (2020). Effect of alternate-day fasting on obesity and cardiometabolic risk: A systematic review and meta-analysis. Metabolism.

[30] Waldman H.S., Renteria L.I., McAllister M.J. (2020). Time-restricted feeding for the prevention of cardiometabolic diseases in high-stress occupations: A mechanistic review. Nutr. Rev..

[31] Wang X., Yan Q., Liao Q., Li M., Zhang P., Santos H.O., Kord-Varkaneh H., Abshirini M. (2020). Effects of intermittent fasting diets on plasma concentrations of inflammatory biomarkers: A systematic review and meta-analysis of randomized controlled trials: Fasting and inflammation. Nutrition.

[32] Meng H., Zhu L., Kord-Varkaneh H., Santos H.O., Tinsley G.M., Fu P. (2020). Effects of intermittent fasting and energy-restricted diets on lipid profile: A systematic review and meta-analysis. Nutrition.

[33] Pureza I.R.O.M., Macena M.L., da Silva Junior A.E., Praxedes D.R.S., Vasconcelos L.G.L., Bueno N.B. (2021). Effect of early time-restricted feeding on the metabolic profile of adults with excess weight: A systematic review with meta-analysis. Clin. Nutr..

[34] Asif S., Morrow N.M., Mulvihill E.E., Kim K. (2020). Understanding dietary intervention-mediated epigenetic modifications in metabolic diseases. Front. Genet..

[35] Adafer R., Messaadi W., Meddahi M., Patey A., Haderbache A., Bayen S., Messaadi N. (2020). Food timing, circadian rhythm and chrononutrition: A systematic review of time-restricted eating’s effects on human health. Nutrients.

